# Mucosa-Associated *Escherichia coli* in Colorectal Cancer Patients and Control Subjects: Variations in the Prevalence and Attributing Features

**DOI:** 10.1155/2021/2131787

**Published:** 2021-11-09

**Authors:** Roghayeh Nouri, Alka Hasani, Kourosh Masnadi Shirazi, Mohammad Reza Alivand, Bita Sepehri, Simin Sotoudeh, Fatemeh Hemmati, Afshin Fattahzadeh, Babak Abdinia, Mohammad Ahangarzadeh Rezaee

**Affiliations:** ^1^Student Research Committee, Tabriz University of Medical Sciences, Tabriz, Iran; ^2^Infectious and Tropical Diseases Research Center, Tabriz University of Medical Sciences, Tabriz, Iran; ^3^Department of Medical Microbiology, Faculty of Medicine, Tabriz University of Medical Sciences, Tabriz, Iran; ^4^Liver and Gastrointestinal Diseases Research Center, Tabriz University of Medical Sciences, Tabriz, Iran; ^5^Department of Medical Genetics, Faculty of Medicine, Tabriz University of Medical Sciences, Tabriz, Iran; ^6^Pediatric Health Research Center, Tabriz University of Medical Sciences, Tabriz, Iran; ^7^Clinical Research Development Unit of Children Educational, Research and Treatment Center, Tabriz University of Medical Sciences, Tabriz, Iran

## Abstract

Accumulating evidence indicates that specific strains of mucosa-associated *Escherichia coli* (*E. coli*) can influence the development of colorectal carcinoma. This study aimed to investigate the prevalence and characterization of mucosa-associated *E. coli* obtained from the colorectal cancer (CRC) patients and control group. At two referral university-affiliated hospitals in northwest Iran, 100 patients, 50 with CRC and 50 without, were studied over the course of a year. Fresh biopsy specimens were used to identify mucosa-associated *E. coli* isolates after dithiothreitol mucolysis. To classify the *E. coli* strains, ten colonies per sample were typed using enterobacterial repetitive intergenic consensus-based PCR (ERIC-PCR). The strains were classified into phylogroups using the quadruplex PCR method. The PCR method was used to examine for the presence of cyclomodulin, *bfp*, *stx*1, *stx*2, and *eae*-encoding genes. The strains were tested for biofilm formation using the microtiter plate assay. CRC patients had more mucosa-associated *E. coli* than the control group (*p* < 0.05). Enteropathogenic *Escherichia coli* (EPEC) was also found in 23% of CRC strains and 7.1% of control strains (*p* < 0.05). Phylogroup A was predominant in control group specimens, while *E. coli* isolates from CRC patients belonged most frequently to phylogroups D and B2. Furthermore, the frequency of cyclomodulin-encoding genes in the CRC patients was significantly higher than the control group. Around 36.9% of *E. coli* strains from CRC samples were able to form biofilms, compared to 16.6% *E. coli* strains from the control group (*p* < 0.05). Noticeably, cyclomodulin-positive strains were more likely to form biofilm in comparison to cyclomodulin-negative strains (*p* < 0.05). In conclusion, mucosa-associated *E. coli* especially cyclomodulin-positive isolates from B2 and D phylogroups possessing biofilm-producing capacity colonize the gut mucosa of CRC patients.

## 1. Introduction

Colorectal cancer (CRC) is the world's third most common cancer and the second leading cause of cancer death [1]. Because of its high morbidity and mortality rate, CRC is an important public health issue [2]. Mutations that occur in tumor suppressor genes, oncogenes, and genes related to DNA repair mechanisms can lead to CRC. Depending on the origin of the mutations, colorectal carcinomas are divided into three types: sporadic (70%), familial (25%), and inherited (5%) [3].

The human gut microbiota contains over more than 1,000 microbial species, adding together 10^14^ microorganisms [4] that play an essential role in many important physiological processes, such as food digestion, metabolism, and immune response [5]. Shifts in the composition of these resident microbiota, the so called microbial dysbiosis, have been found to cause various diseases, including CRC [6, 7], cardiovascular disease, inflammatory bowel disease, and diabetes mellitus [6]. Approximately 20% of all cancers in humans can be related to infectious agents. Gut bacteria may be involved in the initiation or progression of sporadic CRC via a variety of mechanisms, including inducing inflammation, generating reactive oxygen species, and producing genotoxins [8]. Published literature suggests that some bacterial species such as *Bacteroides fragilis* [9], *Streptococcus bovis*, *Fusobacterium* spp., *Helicobacter pylori*, and *Clostridium septicum* are associated with colorectal carcinogenesis [10]. Moreover, considering the fact that *E. coli* is the most common facultative anaerobic resident in human gut flora, several research studies have shown a strong link between mucosa-associated *E. coli* and CRC [8, 11–13]. Moreover, *E. coli* strains are categorized into phylogenetic groups (A, B1, B2, C, D, E, and F) based on the existence or absence of a variety of genes and DNA fragment [14]. Pathogenic *E. coli* strains are mostly found in the B2 or D phylogroups, while commensal strains are mostly found in groups A and B1 [15].

Interestingly, enteropathogenic *Escherichia coli* (EPEC) is commonly found in CRC patients, in contrast to their occasional presence in the control subjects [16]. Additionally, *E. coli* isolates that carry the *eae* gene (encoding for intimin protein) are able to attach the intestinal epithelium and are classed as attaching and effacing *E. coli* (AEEC). More noteworthy, EPEC is also the most widely found AEEC bacteria in humans. This bacterium suppresses the expression of key DNA mismatch repair proteins (MMR), which suggests that chronic mucosal EPEC infection can contribute to the development of CRC tumors [17]. Typical EPEC strains (tEPEC) have the *eae* and *bfp* (gene encoding for bundle-forming pili) genes but no Shiga toxin genes (*stx*). Often known as atypical EPEC, certain clinical isolates of EPEC lack the *bfp* gene [18].

Usually, pathogenic *E. coli* strains produce several toxins called cyclomodulins including the cytotoxic necrotizing factor (CNF), cycle inhibiting factor (Cif), colibactin, and cytolethal distending toxins (CDTs). The colibactin is a toxin produced by polyketide synthetase (*pks*) [8]. Cyclomodulins are attracting growing attention due to their ability to influence cellular differentiation, apoptosis, and cell proliferation by disrupting the eukaryotic cell cycle and/or promoting DNA damage [19]. For example, B2 *E. coli* strains carrying the cyclomodulin-encoding genes are more prevalent in colon tumor biopsies in comparison to control samples suggesting a possible role of these isolates in CRC carcinogenesis [12, 20].

In addition, recent studies have shown that bacterial biofilms are associated with human colon cancer [21, 22]. Mucus-invasive bacterial biofilms, for example, were found on the colon mucosa of CRC patients more frequently than in healthy subjects [23]. Biofilm formation causes an increase in epithelial junction permeability in both normal and cancerous colons, which can facilitate direct access to mutagens from bacteria to the colonic epithelial surface and encourage procarcinogenic tissue inflammation [24]. In general, *E. coli* appears to play a significant role in CRC carcinogenesis, but there are little details on the characterization of colonic mucosa-associated *E. coli* from CRC patients. Therefore, the aim of this analysis was to compare the presence and characteristics of mucosa-associated *E. coli* isolates in biopsy specimens from CRC patients and a control group.

## 2. Materials and Methods

### 2.1. Patients

Between June 2019 and June 2020, 100 patients were examined at two referral university-affiliated hospitals in northwest Iran (Table 1). Among these, 50 patients had CRC, while equal number of patients without CRC had piles (*n* = 26), pruritus ani (*n* = 12), irritable bowel syndrome (IBS) (*n* = 2), and no symptoms (refer for screening; *n* = 10) were taken as controls into the study.

Clinical features (such as weight loss, rectal bleeding, abdominal pain, and bowel habit changes), a positive fecal occult blood test, colonoscopy, and pathology findings were used to diagnose CRC. Furthermore, IBS was diagnosed based on the presence of clinical indications and symptoms (Rome criteria).

In this study, noncancerous individuals without acute or chronic inflammation were included in the control group. In addition, inclusion criteria for CRC patients were a histological diagnosis of malignant tumor without any previous treatment. Moreover, patients who had taken antibiotics in the four weeks leading up to the endoscopy were also excluded from the study.

### 2.2. Sample Treatment

During colonoscopy, biopsy specimens were obtained from the colon and rectum using regular endoscopic forceps. The neoplastic characteristics of the biopsy samples were verified by pathological results. The mucosal biopsy specimens were placed in an Eppendorf tube containing normal saline and transferred to the lab [16]. The samples were washed three times in 5 mL PBS to remove the fecal bacteria [12]. Then, in order to remove the mucus layer, fresh biopsy specimens were transferred to a microtube containing 500 *µ*L of 0.016% wt/vol dithiothreitol solution and rotated for 10 minutes [25]. The obtained supernatant was inoculated in nutrient broth (NB) and incubated at 37°C for 24 hours. After the specified period, broth culture was subcultured on MacConkey agar. *E. coli* isolates were identified by standard conventional biochemical tests.

### 2.3. ERIC-PCR Typing and Phylogenetic Classification

Ten colonies of *E. coli* from each positive sample were typed with enterobacterial repetitive intergenic consensus-based PCR (ERIC-PCR) as described by Versalovic et al. (1991) [26]. All *E. coli* strains were then divided to phylogroups based on the presence of the DNA fragment (TSPE4.C2) and *arpA*, *chuA*, *trpA*, and *yjaA* genes, which were detected by the quadruplex PCR method proposed by Clermont et al. [14].

### 2.4. Detection of Cyclomodulin Genes and Other Virulence Factors

The PCR method was performed using primers specific for the cyclomodulin (*cif, cnf1*, *cnf2*, *cnf3, cdtB-I*, *cdtB-II*, *cdtB-III*, *cdtB-IV, cdtB*-*V*, and *pks* genomic island), *clbA, clbQ* [27], *bfp*, *stx*1, *stx*2, and intimin (*eae*)-encoding genes [25].

The template DNA from *E. coli* isolates was extracted using the boiling water method, as described previously [27]. The PCR reaction was carried out in a 50 *µ*L mixture containing 10–100 ng of the template DNA, 0.2 mM each dNTPs, 0.5 pmol of each primer, 3 mM MgCl_2_, and 1.0 U of DNA polymerase (Yekta Tajhiz Azma, Iran), in the corresponding reaction buffer.

### 2.5. Biofilm Production Assay

Biofilm formation assays were carried out by the microtiter plate method, as described before using 2% crystal violet. Optical density (OD) of each stained well was measured at 570 nm. The cutoff OD was defined as three standard deviations above the mean OD of the negative control. TSB medium without bacteria was considered as a negative reagent control. All isolates were classified into no or weak biofilm producers, moderate biofilm producers, or high biofilm producers [28].

### 2.6. Statistical Analysis

In this study, the results were analyzed by the chi-square test or Fisher's exact test using SPSS software for Windows (version 23 SPSS Inc., Chicago, IL, USA). A *p* value less than 0.05 was considered a statistically significant difference.

## 3. Results

### 3.1. *E. coli* Strains in CRC and Control Group Specimens

**Figure 1:**
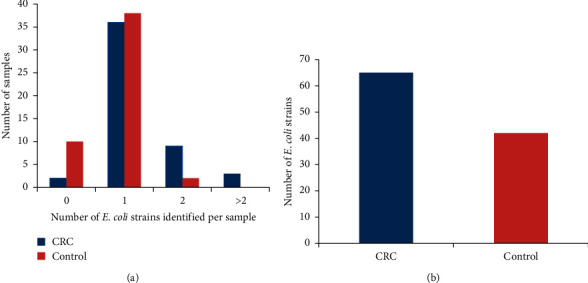
Number of *E. coli* strains obtained from 100 subjects comprising 50 CRC patients and the equivalent number of control subjects. (a) Some specimens did not carry any strain of *E. coli* and some specimens carried one or more than one *E. coli* strains. (b) A total of 65 *E. coli* strains from CRC patients and 42 *E. coli* strains from the control group identified.

The investigation of the presence of *E. coli* isolates showed that the number of specimen without *E. coli* was remarkably higher in control specimens (20%, *n* = 10/50) than in those with CRC patients (4%, *n* = 2/50), *P* = 0.028. In this study, some specimens carried more than one *E. coli* strains. As some specimens carried more than one *E. coli* strains, a total of 65 *E. coli* strains from CRC patients and 42 *E. coli* strains from the control group were taken into the study (Figure 1).

### 3.2. Prevalence of EPEC in CRC and Control Group Samples

EPEC are defined as isolates that have the *eae* and *bfp* genes but no Shiga toxin genes. Often known as atypical EPEC, certain clinical isolates of EPEC lack the *bfp* gene.

**Figure 2:**
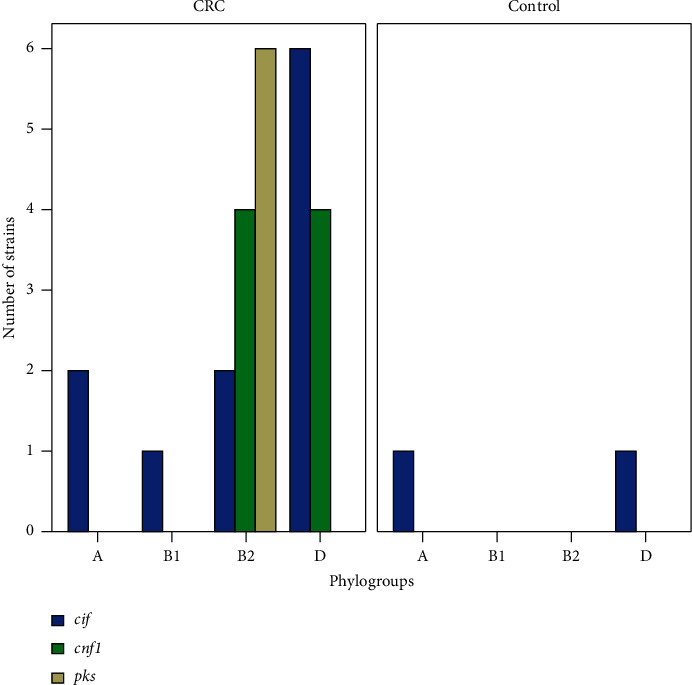
Distribution of cyclomodulin-positive *E. coli* in various phylogenetic groups.

The *eae* gene was detected in 23% *E. coli* isolated from clinical specimen obtained from the CRC group, but only in 7.1% *E. coli* isolated from the control group, the difference being statistically significant (*p*=0.036). Moreover, all *eae*-positive strains were negative for *stx*1 and *stx*2 genes. Interestingly, all EPEC strains were atypical EPEC (PCR negative for bfp) (Figure 2).

### 3.3. Distribution of *E. coli* Phylogroups according to the Specimen Origin

Phylogenetic studies revealed that the prevalence of phylogroups in *E. coli* strains from CRC specimens and controls varied significantly. As given in Table 2, phylogroup D was the predominant type in *E. coli* obtained from CRC patients (41.5%), followed by phylogroups B2 (36.9%), A (13.9%), and B1 (7.7%). On the other hand, *E. coli* isolated in the control group most frequently belonged to phylogroup A (38.1%), followed by phylogroups B1 (19%), D (16.7%), B2 (11.9%), E (9.5%), and F (4.7%).

### 3.4. Distribution of Cyclomodulin-Encoding Genes according to the Sample Origin and Phylogroups

As given in Table 3, the increased frequencies of cyclomodulin-encoding genes on CRC samples (30.7%, *n* = 20/65) compared to control tissues were statistically significant (4.7%, *n* = 2/42), *p*=0.001. It is worth noting that the *cif* gene was the only gene revealed (4.7%) among cyclomodulin-encoding genes in *E. coli* strains obtained from the control group. Among *E. coli* strains from CRC patients, the strains harboring *cif, cnf1*, and *pks* genes represented 16.9%, 12.3%, and 9.2% of the total strains isolated, respectively. Additionally, 7.7% (*n* = 5) *E. coli* strains from CRC patients carried more than one cyclomodulin-encoding genes. Moreover, *cnf2*, *cnf3*, and *cdtB* genes were not found in any of the strains from CRC specimens.

All strains of patients in the CRC group harboring *pks* genomic island belonged to phylogroup B2. Moreover, 50% *E. coli* strains in this group harboring *cnf1* gene belonged to phylogroup B2, while 50% *E. coli* strains belonged to phylogroup D. The distribution of phylogroups amongst *E. coli* strains of the CRC group possessing *cif* gene was as follows: 54.5% belonged to phylogroup D, followed by phylogroups A (18.2%), B2 (18.2%), and B1 (9.1%) (Table 3).

### 3.5. Biofilm-Forming Ability of *E. coli* Strains from the Control Group and CRC Specimens

As given in Table 4, in this study, there was a significant difference between the ability to form biofilms in the CRC group (36.92%, *n* = 24/65) and the control group (16.6%, *n* = 7/42), *p*=0.03. Amongst the biofilm-producing strains from the control group, all strains were weak biofilm producers. In contrast, amongst *E. coli* strains of the CRC group, 33.3% were observed as strong or moderate biofilm producers, while 66.6% were weak biofilm producers.

The number of biofilm-positive strains was significantly higher in cyclomodulin-positive *E. coli* strains (60%, *n* = 12/2) compared to cyclomodulin-negative *E. coli* strains (26.6%, *n* = 12/45), *p*=0.014.

## 4. Discussion

CRC is one of the deadliest cancers in the world. Indeed, bacterial infection has long been known as key factor in the etiology of CRC [8]. More recently, accumulating evidence supports that mucosa-associated *E. coli* can affect the development of CRC [8, 29, 30]. However, there are relatively few data available about the prevalence and characterization of mucosa-associated *E. coli* from CRC specimens.

In our study, the colonic biopsy specimens from CRC and control patients indicated that CRC samples are more colonized by mucosa-associated *E. coli* strains in comparison to the control group. This result is consistent with the results of other studies [12, 31].

Additionally, EPEC was found to be more prevalent in CRC (23%) than in control samples in this study (7.1%). Magdy et al. have previously reported the presence of EPEC isolates in about 50% of CRC specimens and 20% healthy control [16]. Furthermore, recent evidence shows that chronic mucosal EPEC infection could promote molecular pathways involved in CRC carcinogenesis [17]. The higher colonization of biopsy specimens of CRC patients by mucosa-associated *E. coli* can be possibly explained that modifications of the colon mucosal properties in CRC lead to increased expression of adhesion and internalization of pathogens in the tumor microenvironment [8].

In the current study, the distribution of *E. coli* phylogroups in CRC patient was different from the control group. Most strains from CRC patients belonged to B2 and D phylogroups, while about 58% of the strains from the control group belonged to A and B1 phylogroups. Thus, it can be concluded that the intestinal mucosa of CRC patients is mainly colonized by *E. coli* strains that are more virulent. Buc et al. [12] and Raisch et al. [11] previously identified higher numbers of *E. coli* phylogroup B2 in CRC specimens than in controls in two studied. However, no major difference in the abundance of *E. coli* belonging to the phylogenetic group D was found between patients with CRC and diverticulosis in their studies. Group B2 *E. coli* strains can cause severe infections because their genetic background is favorable for obtaining the high number of virulence factors [32]. Additionally, macrophages are one of the main tumor-infiltrating immune cells which have a pivotal role in cancer progression. Interestingly, *E. coli* of the B2 phylogroup can survive and multiply within macrophages and promote protumoral activities of macrophages independently of colibactin production [33].

In the current research, CRC patients had a substantially higher frequency of cyclomodulin-positive *E. coli* than the control group, comparable to other studies performed by Bonnet et al. [8], Raisch et al. [11], and Buc et al. [12]. Overall, the findings indicated that cyclomodulin-positive *E. coli* isolates are implicated in the development of CRC.

We observed cyclomodulin-positive *E. coli* strains in only 4.7% of the control samples, while Buc et al. [12] and Bonnet et al. [8] observed these isolates in 19.4% and 26% of control samples, respectively. The high frequency of cyclomodulin-positive *E. coli* in their studies compared to ours may be due to they used diverticulosis patients as a control group and dysbiosis of gut microbiota has been reported in diverticulosis patients [34].

In our study, the prevalence of *pks*-positive *E. coli* isolated from CRC patients was about 10%, which in contrast to previous research studies is significantly lower. Other research studies have reported *pks*-positive *E. coli* isolated in CRC patients to range from 26% to 67% [8, 11, 12, 35]. *pks* *+* *E. coli* isolates are attracting attention because of their ability to induce DNA damage in epithelial cells, leading to genomic instability of mammalian cells [36–40] and thus promote development of colorectal cancer in animal models [35]. Over the course of 30 years, *E. coli* isolates from fecal samples of populations living in industrialized countries showed a phylogenetic shift from group A toward group B2. Moreover, about 30% of this commensal B2 *E. coli* isolates have acquired *pks* island by horizontal transfer [41].

Presence of cyclomodulin-encoding gene *cif* was revealed in 17% *E. coli* strains belonging to the CRC patients group. In addition, 12.3% of *E. coli* strains were positive for *cnf* gene. More noteworthy, amongst cyclomodulin-encoding genes, *cif* was the most common in our study, in contrast to other research studies whereby *pks* has been reported as the most common cyclomodulin-encoding gene [11, 12]. Thus, our study revealed that in addition to *pks*, other cyclomodulins especially *cif* and *cnf* might play an important role in development of CRC.

In this analysis, the number of biofilm-producing *E. coli* strains was higher in CRC samples than in control samples (*p* < 0.05). Furthermore, as compared to *E. coli* strains from healthy mucosa, CRC patients' *E. coli* isolates were high biofilm producers. Interestingly, we also found that cyclomodulin-positive *E. coli* isolates were more likely to generate biofilm than cyclomodulin-negative isolates in the current research. In this regard, Tomkovich et al. have previously reported the presence of mucus-invasive biofilms on the colon mucosa of about half CRC samples and in only 13% of control specimens [23]. In addition, Dejea et al. for the first time displayed that bacterial biofilms are associated with CRC. Presence of biofilms in tissue may correlate with bacterial invasion and changes in colon tissue biology with increased cellular proliferation [42].

The predominance of adhesin-encoding genes, particularly pili adhesins, that encourage colonization of carcinogenic strains in the intestinal tissue or colorectal tumor microenvironment was not detected in this investigation. It also does not examine into the cyclomodulin-producing strains' carcinogenic potential in animal studies.

As a result, we must examine the frequency and expression of adhesin-encoding genes in *E. coli* strains isolated from CRC patients and controls in the near future. In addition, we must investigate the ability of cyclomodulin-producing strains to interact with intestinal epithelial cells, as well as their potential carcinogenic effect in animal models.

## 5. Conclusion

Our study showed that *E. coli* isolates obtained from the CRC patients and controls are a heterogeneous group of isolates with variations in virulence factors, phylogroups, and biofilm-forming capacity. To summarize, CRC samples were more colonized by mucosa-associated *E. coli*, especially cyclomodulin-positive isolates from B2 and D phylogroups with the ability to form biofilms in comparison to the control group. This is the largest study of the prevalence and characterization of mucosa-associated *E. coli* isolated from CRC patients and a control group in Iran that we are aware of.

## Figures and Tables

**Table 1:** Characteristics of CRC patients and control group subjects.

Characteristics	CRC patients (*n* = 50)	Control group (*n* = 50)	*P* value
Age, median (range)	64 (40–83)	56 (31–82)	*P* > 0.05
Gender, male/female	27/23	17/33	*P* > 0.05
BMI (kg/m^2^) (mean ± SD)	25.6 ± 3.1	26.1 ± 3.3	*P* > 0.05

**Table 2:** Phylogroups distribution of *E. coli* isolates according to specimen origins.

Specimen types	Number (%) of *E. coli* strains in various phylogenetic groups					
	A	B1	B2	D	E	F
CRC patients*∗*	9 (13.9)	5 (7.7)	24 (36.9)	27 (41.5)	0 (0)	0 (0)
Control group*∗∗*	16 (38.1)	8 (19)	5 (11.9)	7 (16.7)	4 (9.5)	2 (4.7)

^*∗*^65 *E. coli* strains were obtained from 50 CRC patients; ^*∗∗*^42 *E. coli* strains were obtained from 50 control group subjects.

**Table 3:** Distribution of cyclomodulin-positive *E. coli* according to phylogenetic groups.

Percentage (number) of *E. coli* strains from CRC patients in diverse phylogenetic groups							
	A (*n* = 9)	B1 (*n* = 5)	B2 (*n* = 24)	D (*n* = 27)	E (*n* = 0)	F (*n* = 0)	All (*n* = 65)

*cif*	22.2 (2)	20 (1)	8.3 (2)	22.2 (6)	0 (0)	0 (0)	16.9 (11)
*cnf*_*1*_	0 (0)	0 (0)	16.7 (4)	14.8 (4)	0 (0)	0 (0)	12.3 (8)
*pks*	0 (0)	0 (0)	25 (6)	0 (0)	0 (0)	0 (0)	9.2 (6)
*cdt*	0 (0)	0 (0)	0 (0)	0 (0)	0 (0)	0 (0)	0 (0)
Cyclomodulin-encoding genes	22.2 (2)	20 (1)	50 (12)*∗*	37 (10)*∗*	0 (0)	0 (0)	30.7 (20)*∗*

Percentage (number) of *E. coli* strains from the control group in diverse phylogenetic groups							
	A (*n* = 16)	B1 (*n* = 8)	B2 (*n* = 5)	D (*n* = 7)	E (*n* = 4)	F (*n* = 2)	All (*n* = 42)

*cif*	6.2 (1)	0 (0)	0 (0)	14.3 (1)	0 (0)	0 (0)	4.7 (2)
*cnf*	0 (0)	0 (0)	0 (0)	0 (0)	0 (0)	0 (0)	0 (0)
*pks*	0 (0)	0 (0)	0 (0)	0 (0)	0 (0)	0 (0)	0 (0)
*cdt*	0 (0)	0 (0)	0 (0)	0 (0)	0 (0)	0 (0)	0 (0)
Cyclomodulin-encoding genes	6.2 (1)	0 (0)	0 (0)	14.3 (1)	0 (0)	0 (0)	4.7 (2)

^*∗*^Some *E. coli* isolates carried more than one cyclomodulin-encoding genes.

**Table 4:** Percentage (number) of biofilm-positive isolates and their biofilm formation ability.

	Biofilm-positive strains	Biofilm formation category	
		Moderate/strong producers	Weak producers
Cyclomodulin-positive strains from CRC patients (*n* = 20)	60 (12)	50 (6)	50 (6)
Cyclomodulin-negative strains from CRC patients (*n* = 45)	26.6 (12)	16.6 (2)	83.3 (10)
CRC (*n* = 65)	36.9 (24)	33.3 (8)	66.6 (16)
Cyclomodulin-positive strains from the control group (*n* = 2)	50 (1)	0 (0)	100 (1)
Cyclomodulin-negative strains from the control group (*n* = 40)	15 (6)	0 (0)	100 (6)
Control (*n* = 42)	16.6 (7)	0 (0)	100 (7)

## Data Availability

The authors declare the data used to support the findings of this study are available from the corresponding author upon request.
